# Modelling microbial metabolic rewiring during growth in a complex medium

**DOI:** 10.1186/s12864-016-3311-0

**Published:** 2016-11-24

**Authors:** Marco Fondi, Emanuele Bosi, Luana Presta, Diletta Natoli, Renato Fani

**Affiliations:** Department of Biology, University of Florence, Via Madonna del Piano 6, I-50019 Sesto F.no, Italy

**Keywords:** Flux balance analysis, *Pseudoalteromonas haloplanktis* TAC125, Antarctic bacteria, Metabolic modelling

## Abstract

**Background:**

In their natural environment, bacteria face a wide range of environmental conditions that change over time and that impose continuous rearrangements at all the cellular levels (e.g. gene expression, metabolism). When facing a nutritionally rich environment, for example, microbes first use the preferred compound(s) and only later start metabolizing the other one(s). A systemic re-organization of the overall microbial metabolic network in response to a variation in the composition/concentration of the surrounding nutrients has been suggested, although the range and the entity of such modifications in organisms other than a few model microbes has been scarcely described up to now.

**Results:**

We used multi-step constraint-based metabolic modelling to simulate the growth in a complex medium over several time steps of the Antarctic model organism *Pseudoalteromonas haloplanktis* TAC125. As each of these phases is characterized by a specific set of amino acids to be used as carbon and energy source our modelling framework describes the major consequences of nutrients switching at the system level. The model predicts that a deep metabolic reprogramming might be required to achieve optimal biomass production in different stages of growth (different medium composition), with at least half of the cellular metabolic network involved (more than 50% of the metabolic genes). Additionally, we show that our modelling framework is able to capture metabolic functional association and/or common regulatory features of the genes embedded in our reconstruction (e.g. the presence of common regulatory motifs).

Finally, to explore the possibility of a sub-optimal biomass objective function (i.e. that cells use resources in alternative metabolic processes at the expense of optimal growth) we have implemented a MOMA-based approach (called nutritional-MOMA) and compared the outcomes with those obtained with Flux Balance Analysis (FBA). Growth simulations under this scenario revealed the deep impact of choosing among alternative objective functions on the resulting predictions of fluxes distribution.

**Conclusions:**

Here we provide a time-resolved, systems-level scheme of *Ph*TAC125 metabolic re-wiring as a consequence of carbon source switching in a nutritionally complex medium. Our analyses suggest the presence of a potential efficient metabolic reprogramming machinery to continuously and promptly adapt to this nutritionally changing environment, consistent with adaptation to fast growth in a fairly, but probably inconstant and highly competitive, environment. Also, we show i) how functional partnership and co-regulation features can be predicted by integrating multi-step constraint-based metabolic modelling with fed-batch growth data and ii) that performing simulations under a sub-optimal objective function may lead to different flux distributions in respect to canonical FBA.

**Electronic supplementary material:**

The online version of this article (doi:10.1186/s12864-016-3311-0) contains supplementary material, which is available to authorized users.

## Background

In their natural environment, bacteria are confronted with a wide range of environmental conditions that change over time. It is generally observed that, when facing a nutritionally rich environment, bacteria first use the “preferred” compound(s) (presumably those allowing the fastest growth rate) and only later start metabolizing the other one(s). The decrease in concentration of these compounds corresponds with the start of usage of the others. In this respect, Monod discovered the phenomenon of “diauxie”, i.e. the microbial capability of utilizing the various nutrients regardless of their abundance but regulating their uptake through the modulation of the overall enzymatic state [1, 2]. This kind of switching of metabolic preference is characteristic of systems that optimize fitness [3]. It is usually observed that, as the bacterium changes from one carbon source to another, growth is temporary halted, while a new of enzymes needed to metabolize alternative nutrients are synthesized. This cellular regulation is likely to play a key role, i.e. adjust cellular nutrient fluxes across the entire bacterial metabolic network as to produce the optimal growth rate [4]. In other words, regardless of the C-source that is used in a specific growth phase, the metabolic network of an organism has to continuously and dynamically adjust to optimally sustain cellular growth. Accordingly, the optimal flux distribution achieved by growing cells sometimes changes discontinuously as the composition of the growth medium is varied. Therefore, a small change in nutrient concentration due, for example, to compounds exhaustion, may sometimes induce a large change in the enzymatic composition of the bacterium [3].

Examples of system-level cellular adjustments following changes in nutrients availability have been recently described exploiting –omics technologies [5, 6]. These works revealed presence of a deep and systemic re-organization of the overall microbial metabolic network in response to a variation in the composition/concentration of the surrounding nutrients. The range and the entity of such modifications in organisms other than a few model microbes (i.e. *E. coli*, *S. coelicolor*) are still unknown, despite they might provide interesting hints in the understanding of non-trivial metabolic phenotypes (e.g. lifestyle decisions and virulent phenotypes [7, 8]).

In order to systemically interpret such complex behaviours, scientists have started exploiting computational models [3, 9] and, as in [10], their integration with phenotypic and gene expression data*.* In this context, constraints-based approaches, in particular flux balance analysis (FBA), have been shown to be predictive of growth phenotypes [11, 12] and can be used to construct large scale metabolic models based on genome sequences and to infer the metabolic impact of perturbations in the external conditions (e.g. nutrients depletion) [10].

Although the presence of metabolic switches in other organisms is less documented, a valuable exception is represented by the Antarctic bacterium *Pseudoalteromonas haloplanktis* TAC125 (*Ph*TAC125). *Ph*TAC125 has been isolated from sea water sampled along the Antarctic ice-shell and is considered one of the model organisms of cold-adapted bacteria. It is capable of thriving in a wide temperature range and it has been suggested as an alternative host for the soluble overproduction of heterologous proteins, given its ability to rapidly multiply at low temperatures [13–16]. A genome-scale metabolic reconstruction of *Ph*TAC125 has been recently published by our group [17], allowing a system-level representation of its phenotypic landscape and boosting a holistic comprehension of its metabolic features. Interestingly, the growth curve of *Ph*TAC125 in a complex medium (peptone) is characterised by a number of metabolic switches among the amino acids available in the medium [14]. The progressive exhaustion of the different sets of amino acids (especially glutamate) defines a hierarchical usage of carbon sources and, consequently, multiple phases in its growth curve, characterized by a different set of metabolized substrates. As mentioned previously, this behaviour points towards a (still undisclosed) reprogramming of cellular metabolism, the effects of which cannot be appreciated just looking at the resulting growth curve.

The aim of this study is to systemically investigate the metabolic adjustments that are (computationally) predicted to occur over time in a microbial cell grown in a complex medium, with particular attention to the global effects produced by multiple substrates switching (e.g. percentage of genes involved, network robustness). Constraint-based metabolic modelling was used to simulate microbial growth in a complex medium over several time steps, each characterized by a specific set of amino acids to be used as carbon and energy source. As said, we examined these features in *Ph*TAC125 taking advantage of its recently published genome-scale metabolic reconstruction and the availability of accurate physiological data in complex media. Results obtained from our simulations allow us to hypothesize the occurrence of a deep metabolic reprogramming following each transition in the availability of nutrients, with more than one half of its metabolic reactions involved. Interestingly, catabolic pathways are predicted not to be the only processes affected by such changes, with many central pathways that seem to be affected by apparent minor changes in the metabolized substrates. Furthermore, we show that our modelling framework is able to capture possible functional patterns and/or common regulatory features of the genes embedded in our reconstruction (e.g. the presence of common regulatory motifs). Finally, we used our modelling framework to explore the effect of simulating a sub-optimal microbial growth, i.e. accounting for the re-direction of part of the cellular resources in alternative metabolic processes at the expense of optimal growth rates. The MOMA-based [18] approach introduced here (nutritional-MOMA) was compared to FBA-derived predictions, revealing interesting insights into the resulting predictions of fluxes distribution.

## Methods

### Model parameterization

The metabolic model used in this manuscript has been described in Fondi et al. [17] and can be found in Additional file 1, together with the codes used to produce the results reported herein. The physiological data of *Ph*TAC125 growth in soy-peptone complex medium [14] was used to identify time steps corresponding to amino acid depletions. Overall, twelve different (one hour long) time steps were identified along the complete growth curve and, for each of these time steps, the uptake rates of each amino acid were estimated based on their relative decrease in concentration and on the growth rate. More in detail, to fit our model to Wilmes et al. data, first we computed the *Ph*TAC125 yield. To do this, we had to derive the difference in biomass for each time step. First, we retrieved the optical density (OD) and growth rate (μ) at each time (0 to 12 h), deriving the ratio between biomass (g/l) and OD, by dividing the reported weight at the 10 h mark (1.28 g) for the corresponding optical density (3.8) times the initial operation volume of the growth experiment (1.4 l). Then, we computed the difference (delta) in biomass between each hour (from 0 to 11) and the following one (1 to 12 time steps). We also computed, for each amino acid, the mass concentration (g/l) at each time step from the corresponding molar concentration values. Finally, we computed for each amino acid the difference in concentration (delta) for each time step, considering this value as 0 when a concentration was increasing from an hour to the following one (nutrient accumulation). Thus, for each time step, the biomass delta value represents the relative increase in biomass, while the sum of the amino acid delta values (one for each amino acid consumed by *Ph*TAC125) represents the total concentration of carbon consumed in that time step. The ratio of these values allowed us to compute the yield value corresponding to each time step.

To allow for growth simulations consistent with the experiment by Wilmes et al. [14], we also had to compute the uptake fluxes (UF) for each amino acid at each time step. To do this, we used the following formula:

$$ {\mathrm{UF}}_{\mathrm{a}\mathrm{a}}^{\mathrm{i}}=\frac{{\mathrm{conc}}_{\mathrm{a}\mathrm{a}}^{\mathrm{i}}}{{\displaystyle {\sum}_{\forall \mathrm{a}\in \mathrm{AA}}}{\mathrm{conc}}_{\mathrm{a}}^{\mathrm{i}}}\ *\frac{{\mathrm{GR}}^{\mathrm{i}}}{{\mathrm{yield}}^{\mathrm{i}}}*\frac{1}{{\mathrm{MW}}_{\mathrm{a}\mathrm{a}}\ *\ {10}^{-3}\ } $$

Where: UF_aa_^i^ is the UF (mmol * g^−1^ * h^−1^) of the amino acid *aa* (belonging to the set of amino acids *AA*) in the time step *i*; GR^i^ and yield^i^ are the *Ph*TAC125 growth rate and yield, respectively, at the time step *i*; conc_aa_^i^ and MW_aa_ are the concentration (g/l) and molar weight values, respectively, of the amino acid *aa* in the time step *i*; ∑_∀ a ∈ AA_conc_a_^i^ is the summation of each amino acid concentration in the time step *i*. Finally, we multiplied each uptake flux for a constant specific to the corresponding time step to allow for comparable flux values for each time step.

*In silico* growth was simulated for each phase using FBA (see below) with salts uptake as defined as in Fondi et al. [17] and adding amino acid uptake reactions with lower bound equal to the uptake rate estimated from growth data as described above.

### Modelling procedures

Flux Balance Analysis (FBA) was employed to simulate fluxes distribution at each different time step [19]. As during the last two time steps there was no growth and no amino acids uptake, we limited our analysis to the first ten time steps identified as previously described. The reconstructed model was analysed using COBRAToolbox-2.0.6 [20] in MATLAB® R2012b (Mathworks Inc.). Gurobi 5.6 (www.gurobi.com) solver was used for computational simulations presented herein. The COBRApy package was also used during model expansion and preliminary growth simulations [21]. Statistical analyses on the predicted fluxes were performed using the R package [22].

Flux Variability Analysis (FVA) was performed using the COBRA toolbox. The ratio between maximum and minimum admissible flux of each reaction (v_*i*_) in each of the growth phases was computed as:

$$ {\mathrm{v}}_i={\mathrm{f}}_{max,i}/{\mathrm{f}}_{min,i} $$

with f_*max,i*_ and f_*min.i*_ representing the maximum and minimum admissible fluxes of the *i* ^th^ reaction according to FVA analysis, respectively. On the basis of these spans, we determine the fixed (v_*i*_ = 1) and flexible (v_*i*_ ≠ 1) parts of the metabolic network while achieving a particular metabolic objective (i.e. the biomass formation).

### Identification of co-varying reactions and functional association among their genes

To identify co-varying reactions, we first removed reactions showing a constant trend throughout the simulation. Then, for each of the remaining reactions, we computed the difference (d) between the absolute values of the fluxes following each transition. Formally:

$$ {\mathrm{d}}_z=\left|{{\mathrm{f}}_{j,i}}_{+1}\right|{\textstyle -}\left|{\mathrm{f}}_{j,i}\right| $$

with f_*j,i*_ representing the flux of the *j* ^th^ reaction of the model in the *i* ^th^ time step and d_*z*_ the difference of such fluxes in the *z* ^th^ transition. As we divided the growth of *Ph*TAC125 on complex medium in 10 different phases, for each reaction a vector embedding nine values of d was obtained. Pearson correlation was then computed for each pair of reactions. Finally, we extracted groups co-varying reactions by selecting those reactions sharing a Pearson correlation value greater than 0.7. The sequences of these gene groups were queried to the STRING database [23] to retrieve their possible functional association(s) using its Advanced Programming Inter-face (API). STRING combined score includes in a single value (ranging from 0 to 999) different hints of functional association (such as gene co-expression, co-occurrence, fusion, etc.) among those genes or among their orthologs in organisms other than the selected one (see [24] for details).

### Nutritional-MOMA

With nutritional-MOMA we refer to the use of MOMA [18] optimization to minimize the metabolic adjustments required at each (metabolic) transition of the entire growth period analysed. In its canonical formulation, the MOMA algorithm requires two models, the so called “wild-type” model and the “mutant” model. MOMA can then be used to determine the flux distribution for the “mutant” model that minimizes the difference between the “mutant” model itself and the wild-type solution. We have modified this formulation using, for each transition T among a given growth phase P and the previous one (P-1), the model of phase P as the “mutant” model and the model of phase P-1 as the wild type model. The two models will different in the set of nutrients available during the computation of the flux distribution. Accordingly, this approach should provide a solution in which fluxes are computed in such a way that a minimal metabolic adjustment is computed when the model is presented to a different set of nutrients. For sake of clarity, we named this alternative use of MOMA as nutritional-MOMA. It is to be noticed that, as data from a T0 point (i.e. phase P0) were not available, our nutritional-MOMA computations started from phase P2 (i.e. started from minimizing the metabolic adjustment in the transition between P1 and P2). As a result, plots for this set of analyses embed nine time points rather than ten as in FBA simulations.

### Regulons and conserved motifs identification

The RegPrecise database [25] was used to inspect the presence and the structure of putative regulons in *Ph*TAC125. Specific upstream motifs finding was performed using the Meme suite [26]. Genome-wide regulatory motifs searches were performed using the Dminda server [27] on the set of *Ph*TAC125 upstream regions present in the DOOR database [28].

## Results and discussion

### Modelling procedures

We have recently reconstructed a genome-scale metabolic model of *Ph*TAC125, using it for inferring the metabolic adjustments of this bacterium induced by changes in gene expression following a temperature downgrade in this bacterium [17]. Here we used this model to investigate the metabolic rearrangements occurring during growth in a nutritionally rich medium. A detailed analysis of the data reported by Wilmes et al. [14] allowed the identification of twelve distinct phases in the growth of *Ph*TAC125 on peptone medium, each of them corresponding to a time step of one hour. As during the last couple of phases almost no nutrients uptake was recorded, we limited our analyses to the first ten phases (P1 to P10). For each of these phases we identified the specific uptake rates of the different compounds (amino acids) present in the growth medium (as detailed in Methods) and/or (possible) switches in the use of the available C-sources.

**Fig. 1:**
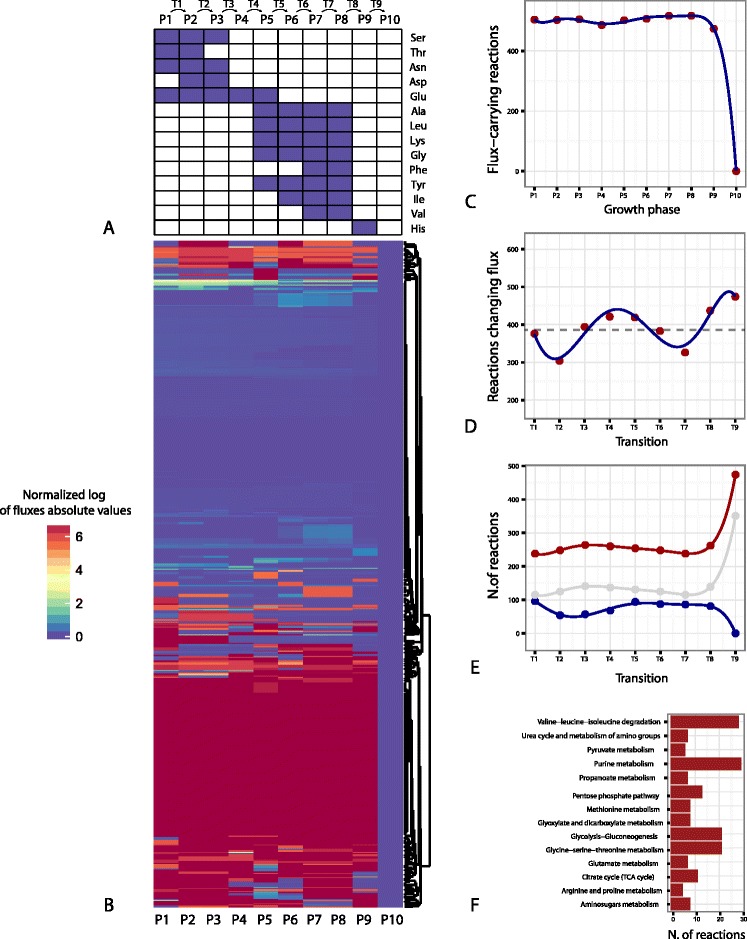
Summary of PhTAC125 genome-scale reprogramming following nutrients switching. **a.** The nutrients provided to the model in each different growth phase according to [14] **b.** Heat map with log values of fluxes across all the phases. **c.** Number of flux carrying reactions in each growth phase. **d.** Number of flux-changing reactions in each growth phase. The dashed line represents the average number of reactions carrying flux over all time points. **e.** Number of reactions whose flux is predicted to increase (*blue line*) and decrease (*red line*) following each shift in the nutrients provided; the black line accounts for those reactions whose flux is predicted to decrease not as an effect of an imposed reduced growth rate during simulations. **f.** functional annotation of reactions varying their flux across all the phases

These values were used as input for ten different FBA simulations (selecting biomass production as the objective function) to derive the most likely fluxes distribution in the *Ph*TAC125 metabolic model in each time step. This allowed a system-level characterization of the metabolic changes occurring after variations in the usage (uptake fluxes) of the different carbon sources. A schematic representation of the nutrients provided to the model in each of the time steps is reported in Fig. 1a.

### Prediction of core and switching reactions

First, we evaluated the predicted flux for each reaction in the model, across all the time points. Overall, we found that 710 reactions did not carry flux in our model in any of the growth phases; these reactions are probably not essential to sustain *Ph*TAC125’s growth in the simulated conditions and were discarded from the following analyses. Conversely, 612 reactions were predicted to carry flux in at least one time point (Fig. 1b) and were considered for further analyses. On average, excluding the last time point in which all the reactions are predicted to be turned off [corresponding to growth rate (*μ*) close to zero], 501 reactions are predicted to carry flux for each of the analysed phases (Fig. 1c). Interestingly, this value is quite constant throughout the time points (standard deviation, s.d. = 13.8, Fig. 1c), revealing that, according to our simulations, the number of reactions necessary to support *Ph*TAC125 growth is predicted to be somehow independent from the carbon sources (amino acids) used as C and energy sources.

Despite this apparently constant trend in the number of metabolic reactions employed by *Ph*TAC125 in a complex medium, the shift among the different growth phases is presumably characterized by a relatively high number of reactions that are predicted (on the basis of our simulation and the biomass-based objective function) to change their flux (Fig. 1d). On average, 392 reactions display at least a single variation in their predicted flux across all the time points (Fig. 1d). However, in this case, a greater variability across all the time points is observed in our simulations (s.d. = 53).

Gene-protein-reaction (GPR) rules of our model indicate that this latter set of reactions is encoded, on average, by 438 genes. Interestingly this number resembles that found when modelling the metabolic switch of *S. coelicolor* (549 enzyme-coding genes, 7% of *S. coelicolor* genes) [10] and represents around 12% of the *Ph*TAC125 coding sequences (55% of the metabolic genes embedded in the metabolic reconstruction).

According to our simulations, switching among the available carbon sources in a complex medium may have an impact on the overall metabolic network of *Ph*TAC125 with more than half of flux-carrying reactions influenced by a change in the utilized C-source or in its uptake rate. More in detail, we found that, on average, 276 reactions are predicted to decrease their flux throughout the growth period (s.d. = 74.8), whereas 69 (s.d. = 30.1) are predicted to increase it. However, the number of reactions whose flux decreases might be biased due to a general decrease, across the time steps, of the growth rate. More specifically, this systemic bias is related to the amino acid uptake rates, which have been derived from the physiological data by Wilmes et al. [14], whose constant decrease lead, for some reactions, to a flux reduction in each time step. Thus, we adjusted the set of “decreasing reactions”, by removing those for which we observed a consistent decrease for all the growth phases. Although the normalization did not affect the general trend, in that the normalized set (grey curve in Fig. 1e) and the not normalized one (red curve in Fig. 1e) have similar trends, the number of decreasing reactions is (for the adjusted set) comparable to that of the increasing reactions. Furthermore, we also computed a normalized flux distribution for each of the modelled growth phases, expressing them as a fraction of the predicted growth rate and evaluated whether this procedure led to different results (in terms of number of reactions carrying and changing flux in each phase and flux increase/decrease patterns). Results (shown and described in Additional file 2: Figure S1) revealed no major differences in the overall trends compared to the original calculation of fluxes distribution, suggesting that our results hold true regardless of the normalization procedure adopted to account for the different (decreasing) growth rates predicted by the model across the growth period.

**Fig. 2:**
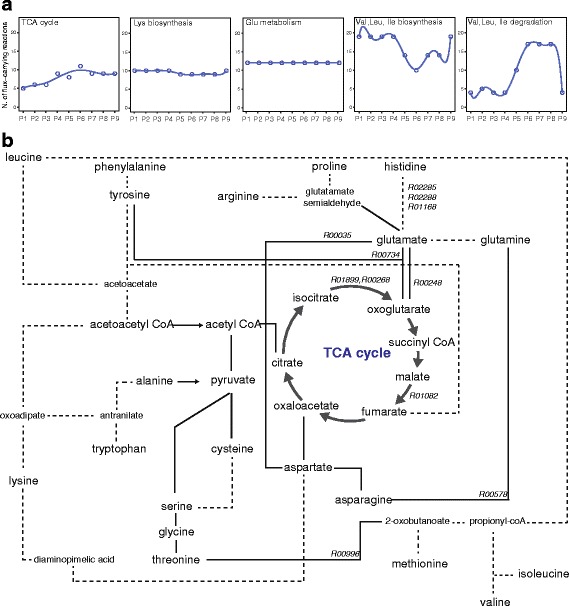
Changes in the central metabolism of PhTAC125. **a.** The number of active (flux-carrying) reactions for five major pathways across all the time points is shown. **b.** A simplified representation of the interconnections in the central metabolism of *Ph*TAC125. Dashed lines indicate the presence of more than one reaction between the connected compounds. Modified from [39]

Not all *Ph*TAC125 metabolic pathways are impacted by this switching of nutrients in the same manner, according to our simulations. Figure 2a shows the hypothetical number of flux-carrying reactions for five pathways, i.e. TCA cycle, Lys biosynthesis, Glu metabolism, Val, Leu and Ile biosynthesis and degradation (a complete overview is presented Additional file 2). TCA cycle, for example, displays and increasing trend in the number of flux-carrying reactions according to our modelling framework; this is consistent with the exhaustion of amino acids (Asp, Asn and Glu) whose degradation provides important TCA cycle intermediates, i.e. oxaloacetate, fumarate and 2-oxo-glutarate and, consequently, with the necessity to activate those reactions leading to the biosynthesis of such compounds. Conversely, Lys and Glu metabolic routes display an overall constant trend (Fig. 2a), with a similar number of active reactions across the different simulated growth phases. This is in line with i) the necessity to use (part) of the lysine biosynthetic route to synthesize diaminopimelic acid, an essential component of bacterial cell wall (see below) and ii) with the importance of Glu metabolism for *Ph*TAC125 (see below and [14]). Finally, Val, Leu and Ile biosynthesis and degradation pathways display an opposite trend one another (Fig. 2a). Intuitively, this reflects the necessity to synthesize these molecules in the first part of the growth phase (when they are not used from the medium) and the necessity to catabolise them once *Ph*TAC125 is using those amino acids as carbon sources, respectively.

We next evaluated whether changes in the kind and number of utilized substrates across all the time points was also parallel to major structural changes at the whole metabolic network level. We used Flux Variability Analysis (FVA) to perform a comprehensive exploration of alternate optimal routes in *Ph*TAC125 metabolic network, in each of the predicted growth phases (as described in Methods). This analysis revealed that the number of “flexible reactions” is predicted to remain constant throughout all the growth phases (data not shown), suggesting that the plasticity of *Ph*TAC125 metabolic network is not particularly dependent on the set of metabolized substrates. This suggests the presence of an efficient adaptation of *Ph*TAC125’s metabolic network to face a nutritionally rich but probably variable environment.

Overall, according to our model, the central phases of the growth curve are characterized by the largest set of reactions changing their flux (T3 to T5 in Fig. 1d), consistently with the major changes in the set of utilized nutrients observed experimentally by Wilmes et al. [14] (Fig. 1a). The first important switch in the panel of utilized compounds occurs between phase 3 and 4 (Fig. 1a). According to experimental evidence from Wilmes et al. [14], in this time point *Ph*TAC125 stops using Ser, Asn and Asp as C and N sources, relying only on glutamate for sustaining growth. The importance of glutamate was previously observed from fed-batch cultivation experiments as it was the most strongly metabolised amino acid in all growth experiments [14]. Also, Glu (together with Pro and Gln) is the amino acid allowing the fastest growth rate (0.11 h^−1^) among all the 20 amino acids when growth in twenty minimal growth media (embedding each amino acid the sole C and N source and an arbitrary uptake rate of 1 mmol * g^(−1)^ * h^(−1)^) was simulated (data not shown).

During P4, *Ph*TAC125 relies only on glutamate for sustaining growth and needs to utilize this compound to derive its main building blocks. L-Glutamate:NADP+ oxidoreductas (KEGG id: R00248) is predicted to be a key reaction in this phase as it allows the conversion of Glutamate to 2-Oxoglutarate to be used as energy source feeding the TCA cycle (Fig. 2b).

More in general, our modelling framework predicts a deep re-programming at the whole metabolic network level following this shift, with 394 reactions changing their predicted flux; more in detail, 264 reactions are predicted to decrease their flux (124 if we exclude those whose decrease may be due to the reduced growth rate imposed to the model for the fitting with experimental values), whereas 57 are predicted to carry an increased flux (Fig. 1e). The remaining (73) reactions are predicted to operate in the reverse direction compared to the previous growth phase following the switch. Among the first set of reactions, 12 are predicted to be turned on in respect to the previous phase, whereas 31 are predicted to be turned completely off. This latter set of reactions includes, as it might be expected, the transporters of serine, asparagine and aspartate, i.e. those compounds that are no longer available in the medium and that cannot be internalized anymore. The cell faces the absence of some of these compounds by redirecting (part) of the glutamate available in the medium to their synthesis. Indeed, for example, the reaction encoded by L-aspartate:L-glutamine amido-ligase (R00578) leading to the formation of asparagine from Gln (derived from Glu) is predicted to double its flux after this metabolic transition (Fig. 2b). The exhaustion of Asp is probably compensated by reversing reaction R00035 (L-Aspartate2-oxoglutarate aminotransferase), generating Asp from Glu (Fig. 2b). Furthermore, our simulation indicated a drop to no flux in the reaction encoded by threonine dehydratase (R00996) and catalysing the conversion of Thr to production of 2-oxobutanoate (and ammonium), a precursor of Val, Leu and Ile (Fig. 2b). A decrease in the availability of Asp (from which Thr is usually synthesized) might impair the biosynthesis of 2-oxobutanoate following this pathway. Our model predicts that *Ph*TAC125 faces this perturbation by reversing the flux of reaction R00999 (cystathionine gamma-synthase), leading to the production of 2-oxobutanoate (and succinate) from O-Succinyl-L-homoserine.

After the following switch (from P4 to P5, T4), Wilmes et al. observed that *Ph*TAC125 starts using Ala, Leu, Gly and Tyr (together with Glu) (Fig. 1a). According to our modelling framework, this involves a predicted change in the flux of 421 reactions (Fig. 1d), with 260, 68 and 93 of them decreasing, increasing or changing the direction of their flux, respectively. Consistently with this new set of amino acids, the model predicts that most of the biosynthetic routes leading to the production of such compounds result turned “off”. This is the case of the path leading to the synthesis of Leu in the branched-chain amino acid biosynthetic route (from 3-isopropylmalate dehydratase to 2-oxoglutarate aminotransferase, R01213, R03968, R01652, R04001, R04426). The same occurs for Ala biosynthesis (accordingly, the reactions catalyzed by L-asparaginase (R00485) and L-Aspartate 4-carboxy-lyase (R00397) are predicted to be turned off) and Lys biosyntheses. Interestingly, in the latter case, only the final step of the whole pathway (Diaminopimelate decarboxylase, R00451) is predicted to carry no flux, consistently with the observation that the rest of the pathway is required for the synthesis of meso-2,6-Diaminopimelate, an essential precursor for peptidoglycan assembly (Fig. 2b).

Amino acid biosynthetic routes are not the only pathways affected by this metabolic switch, according to our simulated growth model. Two TCA cycle reactions are predicted to be turned on following the utilization of Ala, Leu, Gly and Tyr, i.e. those leading to the production of 2-Oxoglutarate from Isocitrate (R01899 and R00268, Fig. 2b). This raises the intriguing question on the source of 2-Oxoglutarate in the previous growth phases given that those TCA reactions were predicted to carry no flux. A possible explanation is provided by the observation that 2-Oxoglutarate can be obtained from the carbon skeletons of several five-carbon amino acids through a first conversion into Glu*,* which is then oxidatively deaminated by glutamate dehydrogenase to yield α-ketoglutarate. However, the presence of Glu in the medium would not favour this solution as the major fraction of Glu can be obtained without additional energy expenses. Alternatively, 2-Oxoglutarate can be obtained from the conversion of Glu (the only carbon source in P4) to Tyr (required for sustaining growth in P4). In our model this reaction (R00734) is predicted to be “on” during P5 thus allowing the synthesis of 2-oxoglutarate (Fig. 2b). However, starting to use Tyr present in the medium during P5 might cause reaction R00734 to be turned off and, consequently, the necessity to synthesize 2-oxoglutarate from succinate to maintain the functioning of TCA cycle. Similarly, the production of fumarate from malate (R01082) is predicted to carry no flux in the shift to P5 (Fig. 2b). Again, this is consistent with the degradation of amino acids as a major source of important metabolic intermediates, as fumarate can be obtained from the degradation of aromatic amino acids as Tyr, available in the medium during P5 and then tunnelled into the TCA cycle.

The time-resolved growth data from Wilmes et al. [14] show that, after other minor transition in which few additional amino acids are degraded (Phe, Ile, Val), the final switch involved the utilization of histidine as the only C source and causes a major drop in the growth rate of *Ph*TAC125 [14]. In our model, this corresponds to the highest number of flux-changing reactions (with the exclusion of the last time point in which all the reactions are turned off) and thus, to the deepest reprogramming encountered by *Ph*TAC125 during this growth curve.

As it might be expected, histidine biosynthetic reactions (R03013, R01163, R03012, R04035, R04640, R03457, R03243, R01071, R04558, and R04037) are predicted to carry zero flux following this transition. Despite the entire pathway is predicted to carry no flux, AICAR (1-(5'-Phosphoribosyl)-5-amino-4-imidazolecarboxamide), one of the intermediates of histidine biosynthesis and a crucial precursor in purine metabolism, might still be synthesized through reaction R04559 (adenylosuccinate lyase). This compound, thanks to the flux predicted to be carried in this phase by reaction R01049, leads to the synthesis of 5-Phospho-alpha-D-ribose 1-diphosphate from D-Ribose 5-phosphate.

Furthermore, the switch to the utilization of His as the sole C-source is predicted to be responsible for the stop of the catabolic routes of the previously degraded amino acids (e.g. Ala, Leu, Lys, Gly, Phe, Ile, Tyr) and, consequently, in the stop of the production of important cellular intermediates (such as 2-oxoglutarate, fumarate). According to our simulation, this causes the re-activation of key metabolic reactions that cannot rely on many degradation intermediates as in the previous phase. These include those belonging to the TCA cycle (e.g. Isocitrate dehydrogenase and Fumarate hydratase, R00268 and R01082, respectively) and purine metabolism (R01049, see before).

Finally, the reactions involved in the conversion (degradation) of His into Glu (Formiminoglutamase, Imidazolonepropionase, Histidine ammonia-lyase, R02285, R02288, R01168, respectively) are predicted to be turned on following this final transition, allowing the production of glutamate and, from these, all the major pathways found to carry flux also in phase P4 (Fig. 2b).

### A sub-optimal objective function predicts alternative fluxes distribution

It is worth noticing that all the simulations described up to now were conducted under the FBA canonical assumption of biomass optimality, i.e. assuming that all metabolic fluxes in the cell are geared towards the production of biomass in each moment of the growth curve. However, situations in which cells invest substantial resources in alternative metabolic processes at the expense of optimal growth rates have been analysed and described quite extensively. According to this scenario, cells may invest substantial (energy) resources in a specific metabolic process at the expense of optimal growth, this being reflected by sub-optimal flux distributions [12, 29, 30].

This situation might be observed, for example, when microbes allocate energetic resources for anticipating changing environmental conditions at the expense of optimal growth [31]. Also, when cells are exposed to nutrients fluctuations they might respond with a minimal metabolic adjustment, to avoid the waste of protein synthesis and degradation necessary to reprogram the entire metabolic network and to simultaneously achieve two objectives, i.e. rapid and minimal adjustments. This latter scenario may indeed resemble the actual competition for nutrients that emerges in natural environments and that has been proposed to be reflected by the sequential uptake of nutrients [32].

To explore this alternative scenario, we have accounted for the possibility that the *Ph*TAC125 biomass objective function could not be fully optimized but, instead, in a near-optimal or sub-optimal state. Since FBA classically assumes the optimization of the biomass production flux to compute the most likely fluxes distribution inside the cell, it cannot *per se* provide hints concerning alternative (sub-optimal) fluxes distribution. For this reason, we have used MOMA [18] to study the hypothetical fluxes distribution when minimizing the metabolic adjustments required at each (metabolic) transition of the entire growth period analysed (“nutritional-MOMA”, see Methods). This differs from the canonical formulation of MOMA in which the effects of a gene knock-out are evaluated by providing an approximate solution for a sub-optimal growth flux state (the mutant strain), nearest in flux distribution to the unperturbed state (wild type strain).

Applying this modelling strategy to our study case revealed that the choice of the optimization criterion (i.e. biomass vs. minimal adjustment following nutrients-switching) has a great influence on the predicted fluxes distribution, both in terms of the fraction of the entire network required to sustain *Ph*TAC125 growth and on the set of active pathways.

**Fig. 3:**
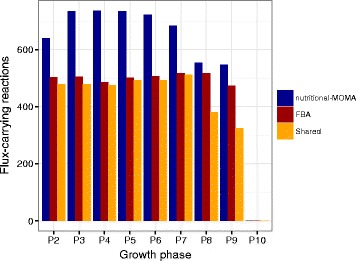
Comparison between nutritional-MOMA and FBA. Here we show the number of predicted flux carrying reactions in each growth phase for FBA (*red*) and nutritional-MOMA (*blue*) optimization on the *Ph*TAC125 model. Also, the number of shared reactions identified by the two approaches is shown (in *yellow*)

First, a higher number of active reactions compared to the original FBA predictions were predicted for each of the growth phases, when the implemented nutritional-MOMA approach was used (Fig. 3 and Additional file 2: Figure S2). This gap is even more evident in the first seven growth phases, whereas the difference between the two approaches becomes negligible in the last three phases. However, despite the two approaches predict a similar number of active reactions in these two final time points, the two sets of reactions appear to be quite different (as shown by the number of shared reactions reported in Fig. 3).

The fact that nutritional-MOMA always predicts a higher number of active reactions might be explained by considering that to optimize the model using FBA identifies the flux distribution maximizing the objective function (biomass optimization), regardless of the metabolic states in the previous time steps. Conversely, nutritional-MOMA will seek for the solution (fluxes distribution) that is closer to the one of each previous time step (i.e., the previous nutritional condition). Therefore, the results of MOMA will likely include a higher number of reactions in the model to produce biomass, since it will minimize the changes (i.e. the active reactions) with respect to the previous time steps, while activating novel reactions to cope with the changes in the nutrient composition.

**Fig. 4:**
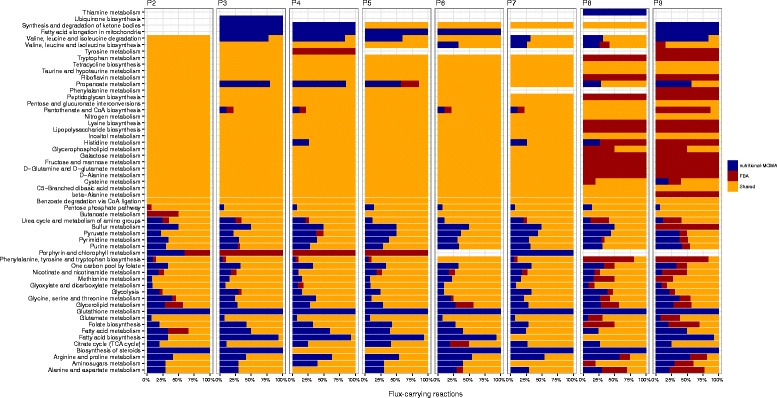
Functional differences between nutritional-MOMA and FBA predictions. Here we show the proportion of reactions predicted to be active by nutritional-MOMA (*blue*), FBA (*red*) and both methods (*yellow*) for each main functional category represented in the *Ph*TAC125 reconstruction

We then investigated whether these differences in the sets of predicted flux-carrying (active) reactions between the two approaches were involved in specific pathways or, rather, spanned over a larger part of the entire *Ph*TAC125 metabolic network. Thus, for each metabolic pathway in the model, we computed the fraction of reactions predicted to be active by nutritional-MOMA, FBA and both methods (Fig. 4). Results of this analysis revealed that the choice of the optimization method impacts the predicted fluxes distribution not only for what concerns the activity of the peripheral (degradation) pathways, i.e. those pathways that start the degradation of amino acids and then tunnel them into the central metabolism. Indeed, we found many central processes (e.g. TCA cycle, Glycolysis, Fatty acids metabolism) in which the proportion of active reactions is (more or less) specific for each of the two optimization strategy (Fig. 4). Notably, for the first six growth phases, most of the reactions predicted to be active under FBA optimizations (and previously described) are carrying flux also adopting the nutritional-MOMA approach. In some cases, however, entire pathways are differently predicted to be active/inactive by the two approaches (e.g. glutathione and tyrosine metabolism in growth phase 3 (Fig. 4). The last two phases of the growth nutritional-MOMA and FBA predict a similar number of active reactions but, from a functional viewpoint, deep differences exist in that entire pathways are predicted to be active only under a specific optimization method (either nutritional-MOMA or FBA, Fig. 4).

**Fig. 5:**
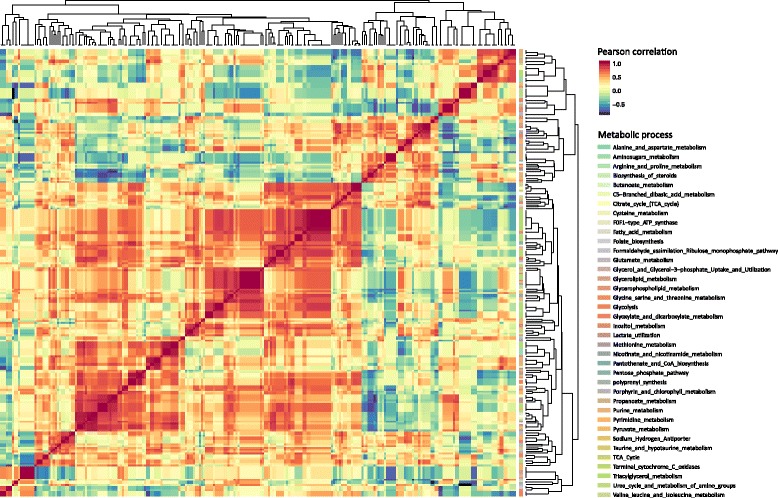
Flux correlation analysis. Heatmap accounting for the Pearson correlation of all the flux difference vectors across all the time points. The metabolic process of each reaction is also reported

To summarize, using our MOMA-based approach we identified a higher number of reactions predicted to be carrying flux in respect to the FBA-based optimization. The nutritional-MOMA approach predicted larger active metabolic networks throughout the analysed phases possessing, on average, about 150 flux-carrying reactions more than the one simulated with FBA. Furthermore, despite most of the active reactions identified through FBA were also identified by the nutritional-MOMA approach, in some cases these predictions differed significantly from a functional viewpoint (as shown in Fig. 5). At present, further experimental evidences are needed to shed light on the real number and function of reactions used by *Ph*TAC125 in each time point (i.e. using each particular nutrients set) and, in other words, to infer how far from the actual fluxes distribution our *in silico* predictions are.

### Flux correlation analysis identifies functionally associated genes

In this section we analyse more in depth the flux matrix (schematically represented by the heatmap of Fig. 1b). This matrix contains the FBA-predicted flux of the reactions (rows) for each of the time steps (columns). As such, it allows capturing the co-variation of reactions in the model; in other words, we identified as paired reactions those whose predicted fluxes displayed a similar trend throughout the time points. These, in turn, may represent functional partners in the cell and/or may belong to the same metabolic pathway/module.

We represented co-varying reactions (sharing flux Pearson correlation ≥ 0.7, see Methods) in the form of a heatmap as shown in Fig. 5. In most cases, the small clusters of reactions showing high Pearson correlation values are involved in the same metabolic process. This is compatible with a scenario in which, following a nutrient(s) switch, entire metabolic pathways are activated (or deactivated) to face the novel environmental condition (as shown in the previous section). Nevertheless, grouping reactions with a lower but still significant threshold (i.e. down to 0.7), clusters start to embed reactions belonging to other metabolic processes. These, in turn, may represent previously undetected functional associations between genes and/or entire pathways.

**Fig. 6:**
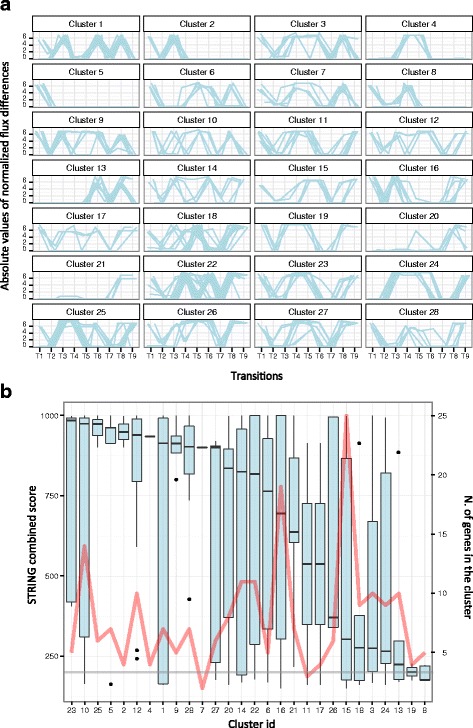
**a**. Co-varying reactions clusters identification. Common flux trends (expressed as the normalized difference between the absolute value of fluxes across each growth phase and the following one) for the reactions embedded in each of the 28 clusters. **b.** The distribution of STRING combined scores among all the genes embedded in each cluster of genes (primary y axis) and the number of genes embedded by each cluster (secondary y axis, red line). The grey line represents the median of the combined score computed for each possible pair of genes in the model

Overall, our method led to the identified 28 different clusters Fig. 6a, comprising 203 reactions. According to the GPR of our model, these reactions were encoded by 223 genes.

We analysed these clusters to answer two biological questions, namely whether the embedded genes i) are *de facto* functionally related and ii) share conserved upstream motifs (which, in turn, would suggest a common regulation mechanism).

#### Groups of co-varying genes display higher combined evidence scores

To address the first point, we exploited the combined evidence score provided by the STRING database. As shown in Fig. 6b, the median of the combined score for the genes embedded in the same cluster is, in most cases, well above the median of all the possible combinations of the genes embedded in the model, suggesting that flux coupling in the model occurs among *bona fide* functional patterns. Accordingly, this result corroborates the capability of our modelling framework to identify functional metabolic modules and the functional associations of their encoding genes in *Ph*TAC125. Interestingly, in some cases apparently un-related genes (showing low combined score values in respect to the other genes) seem to be embedded in the clusters. Despite these instances may represent erroneous predictions of our model, they might also suggest the presence of still undetected functional associations and/or the use of common pools of chemical intermediates by the corresponding reactions (see below).

**Fig. 7:**
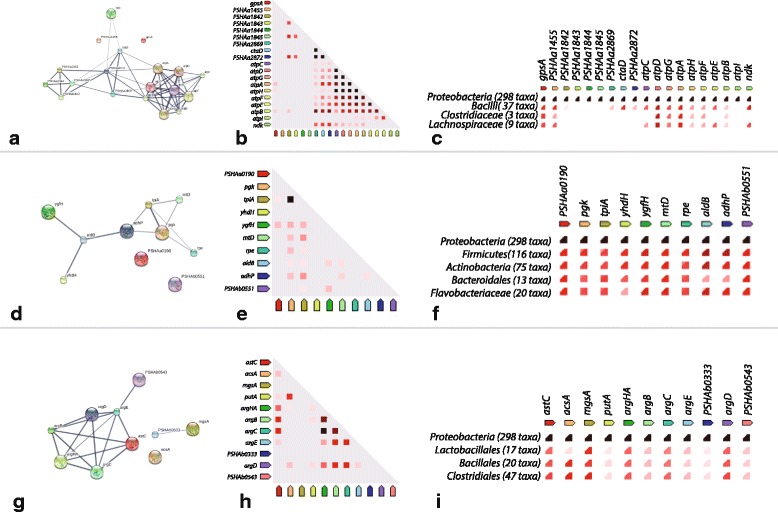
Sample STRING clusters. Evidence network, co-expression instances and co-occurrence patterns for clusters 23 (**a**,**b**, and **c**, respectively), 11 (**d**, **e** and **f**, respectively) and 2 (**g**, **h** and **i**, respectively). Red asterisks in **g** indicate those genes known to belong to the same regulon according to RegPrecise database (ArgR regulon)

We here present a description of the functional relationships retrieved for genes belonging embedded in three of these clusters (Fig. 7). A detailed list of the reactions and genes embedded in all the clusters is provided as Additional file 3 to allow further experimentation and/or *in silico* analyses.

1. **Cluster 23.** This cluster is composed of seven reactions encoded by 19 genes. Three of these reactions are predicted to be catalysed by enzymes encoded by multiple genes, i.e. cytochrome-c oxidase encoded by PSHAa2869-71, Ferrocytochrome-coxygen oxidoreductase encoded by PSHAa1842-45 and ATP synthase encoded by PSHAa3007-15. According to the GPR rules of our model, the simultaneous presence of all the corresponding genes is required to carry flux of each of these reactions. The functional association network obtained probing the STRING database with all the genes of Cluster 23 is reported in Fig. 7a. Genes encoding proteins involved in the same enzymatic reaction appear to be highly interconnected among themselves with evidences that included co-expression, gene fusions, and experimental evidences (see the sub-clusters in the network of Fig. 7a). This is not surprising, as it might be expected that genes encoding proteins that participate in the same enzymatic complex should display coupled regulation and co-occurrence patterns in other organisms. Links, however, are also found among members of different enzymatic complexes, consistent with the fact that these reactions belong to the overall process of oxidative phosphorylation. The *ndk* gene (encoding a nucleoside diphosphate kinase) is also found connected to one of the sub-cluster Fig. 7a). This protein plays a major role in the synthesis of nucleoside triphosphates and is involved in purine and pyrimidine metabolism. The coupling of this reaction’s flux with those belonging to oxidative phosphorylation can thus be explained by their common final products (nucleotide triphosphates and ATP for Ndk and pentose phosphate pathway, respectively), as optimizing the model for biomass production would probably force the flux through both these pathways. Finally, the fact that orthologs of these genes are found to be co-expressed and located in physical proximity in other (related) genomes (Fig. 7b and c, respectively) support the functional association predicted by our model.
2. **Cluster 11.** We found eight reactions (encoded by 10 genes) embedded in this cluster (Fig. 7d), involved in the following metabolic pathways: Glycerolipid metabolism, Pentose phosphate pathway and Glycolysis. Half of the reactions of this cluster are involved in the conversion of glycerol into glycerate 3-pshosphate that, in turn, is connected to glycolysis/gluconeogenesis. As shown by the STRING analysis output, many evidences exist of a functional association among the genes of this cluster, in most cases including instances of co-expression and physical proximity Fig. 7e and f, respectively). Two genes of the cluster remained disconnected in the STRING network (PSHAa0190 and PSHAb0551), both encoding a glycerol kinase and assigned to the same reaction in our metabolic reconstruction (R00847, encoding the transformation of glycerol into sn-Glycerol 3-phosphate). Accordingly, despite no previously detected functional associations were retrieved between these two genes and the other embedded in the same cluster, their presence could be accounted for by the use of common precursors (i.e. glycerol) with the reactions encoded by Cluster 11 genes. Indeed, fluctuations in the availability of such compound (and its derivatives) during the simulated growth transitions explains the coupling of such reactions.
3. **Cluster 2.** This cluster embeds nine reactions encoded by 10 genes involved in Arg biosynthetic pathway *argAH, B, C, D, E* and in the metabolism of Pro (*putA* and Orn cyclodeaminase (PSHAb0543)). The STRING-based evidence network in Fig. 7g revealed hints on the functional associations existing among the genes of the cluster including co-occurrence of orthologs of these genes in other (closely related) genomes, as well as co-expression and conserved genomic neighbourhoods Fig. 7h and i, respectively). The genes embedded in this cluster encode enzymes involved in the the formation of Arg and Pro from Glu, using Asp and then Orn as intermediates. These reactions appear to carry flux especially in the first three phases of the growth (P1 to P3), that is when aspartate (or Asn from which aspartate can be easily obtained) is present in the medium. Afterwards, a decrease in the flux carried by these reactions is observed (the peak in the “Cluster 2” panel of Fig. 7), followed by an overall constant flux trend in the next transitions. Accordingly, the correlation among the fluxes of these reactions could be explained by the need to turn these pathways “on” (or “off”) when glutamate and required intermediates are present (or absent) in the growth medium, respectively. A gene encoding an acetyl-CoA synthetase (*acsA*) is also embedded in this cluster, despite it has not connections with arginine and proline metabolism-related genes. However, its presence might reflect the necessity to synthesize acetyl-CoA from acetate during the first four growth phases identified, i.e. in the absence of ketogenic amino acids. Once *Ph*TAC125 starts metabolising the second set of amino acids, acetyl-CoA can be obtained from the degradation of specific amino acids (e.g. Leu, Val, Ile) and thus the reaction encoded by *acsA* (PSHAa0698) is predicted to stop carrying flux.

#### Analysis of upstream sequences identifies putative regulation mechanisms

In the previous section we reported a list of gene sets which are, presumably, functional partners and whose activity is concerted during the different metabolic switches. If this holds, it is reasonable to hypothesize that some of these clusters might embed co-regulated genes that, in turn, may share conserved upstream motifs (implying for common transcriptional regulatory mechanisms). Thus, to address this point we applied an *ad hoc* computational pipeline (implementing tools from the MEME suite) to analyse the upstream regions of the genes (or arrays of genes in case of operons) embedded in each cluster. This resulted in the identification of motifs that are shared in the upstream regions of all the genes for each cluster. Upstream regions were identified using DOOR (Database of prOkariotic OpeRons) [28]. These have been annotated using three different motif databases (i.e. CollecTF [33], PRODORIC [34] and RegTransBase [35]), to identify motifs with putatively related functions and that might be recognized by putatively related transcription factors (TFs). The whole set of annotations has been manually curated in a conservative way to obtain high-confidence motif annotations. Finally, the obtained putative TFs have been cross-validated by comparing their associated functions with those related to the genes embedded in the clusters.

The whole set of data related to the identified motifs and their annotations (before manual curation), for each database, is provided as Additional file 4. On average, we identified for each cluster a large number of annotations according to the different databases (10.9, 8.9 and 29.4 annotations for CollecTF, PRODORIC and RegTransBase, respectively). However, after manual curation, the number of annotations was reduced to 31, divided in 15 clusters (2.1 on average). Most of the remaining annotations (74%) come from Prodoric, while the other databases have a minor contribution of high-quality annotations (16% CollecTF; 10% RegTransBase).

For each cluster, we eventually compared the putative TFs with the functions of the genes embedded in the cluster, using both literature information and the RegPrecise database. With few exceptions that will be described in details, we found no correlation between the putative TFs and the regulation/biological function associated to the genes embedded in the clusters, suggesting that the majority of the co-varying groups share a common regulation but are regulated by a number of different TFs. We are describing below the clusters for which we found agreement between the predicted TFs and the function of the embedded genes (Table 1).

**Table 1:** Main features of the putatively co-regulated clusters found during flux-correlation analysis. In this table, for each cluster, we report its number, the name of the regulator identified, the genes embedded in it and the conserved motif found upstream of its genes

Cluster name	Motif name	Genes	Weblogo
Cluster 2	ArgR	PSHAa0194, PSHAa0698,PSHAa2175, PSHAa2287, PSHAa2290, PSHAa2291, PSHAa2292, PSHAb0333, PSHAb0428, PSHAb0543	
Cluster 3	CcpA	PSHAa0189, PSHAa0609, PSHAa0740, PSHAa1167, PSHAa1648, PSHAa1649, PSHAa1650, PSHAa1651, PSHAa2167, PSHAb0082, PSHAb0345	
Cluster 6	GalR	PSHAa0603, PSHAa0871, PSHAa1364, PSHAa1767, PSHAa2301, PSHAb0295

1. **Cluster 2.** This cluster, as described previously, is involved in the metabolism of Arg and Pro. A search on the RegPrecise regulon database [25] revealed that, at least in the case of Arg, the genes embedded in the cluster of Fig. 7g are part of a common regulon (ArgR regulon). The only exception is represented by *argD* which, according to the RegPrecise database is not part of the arginine regulon in *Ph*TAC125, and the other genes involved in the biosynthesis of Pro. However, both the computational approach described above and a manual search for putative transcription factor binding sites upstream of these genes detected the already known conserved motif shared by the other *arg* genes of the regulon (Table 1). This suggests that the all the genes are part of the arginine regulon in *Ph*TAC125. More in general, this finding confirms that, starting from our modelling outcomes it is possible to infer biologically consistent patterns of gene co-expression.
2. **Cluster 3.** This cluster includes genes from the *sdh* operon (*sdhABCD*), encoding the succinate dehydrogenases system, as well as other enzymes involved in energy conversion, such as other dehydrogenases (*glpD*, *bcd*) and other enzymes with a role in nucleotide modifications (*hpt*, *ushA* and *mazG*). Our computational pipeline predicted two different regulator genes associated with this cluster, i.e. *ccpA* and *psrA* (Table 1). These regulators encode proteins are involved in the carbon and fatty acid metabolism, respectively. Comparing the functional role of the TFs with the cluster annotation, we can easily relate the set of genes encoding dehydrogenases with *ccpA* and *psrA*, which is also confirmed by RegPrecise database. The other three genes are indirectly involved in the oxidative metabolism, in that they are involved in the metabolism of FAD.
3. **Cluster 6.** This cluster includes five genes, with three of them involved in the synthesis of folate (*folP* and two copies of *folK*), while the remaining two encode a galactokinase (*galK*) and a galactose-1-epimerase (*galM*). All these genes are involved in the metabolism of nucleotides, since folate is necessary to synthesize TDP sugars from UDP, while the other genes encode enzymes that are involved in the synthesis of UDP. The analysis of the upstream sequence of these genes revealed a conserved motif similar to the GalR binding site, thus suggesting that these genes are being co-regulated (Table 1).

## Conclusions

Here we have used constraint-based metabolic modelling to provide a time-resolved, systems-level scheme of *Ph*TAC125 metabolic re-programming following nutrients switching in a nutritionally complex medium. Such features have been analysed using the metabolic model and growth data of the Antarctic bacteria *P. haloplanktis* TAC125. Previous experimental tests revealed a number of nutrients switches in this microorganism when grown in a complex medium [14], consistent with the fact that this Antarctic organism is adapted to fast growth in a fairly rich (but probably inconstant and highly competitive) environment (plankton debris) [36]. Indeed, sequential uptake of nutrients is thought to emerge when competition for nutrients is present [32].

Our modelling framework identified the central phase of the growth curve as a probable key reprogramming point, with more than 400 reactions predicted to adjust their flux in these time points. This corresponds (*in vivo*) to the exhaustion of most of the first metabolized C sources (Ser, Thr, Asn and Asp, most likely the preferred ones), a time step in which only Glu is used as the sole C source, and the final part of the growth curve in which *Ph*TAC125 stops using Glu and relies on a completely different set of nutrients. Our model highlights the occurrence of such an adaption and the need for reprogramming a large set of reactions to maintain an efficient metabolic network. A similar scenario is observed at the end of the growth, when His is used as the sole C source. This transition is the most demanding, requiring a change in the predicted flux of almost 450 reactions.

Taken together, our simulation indicates the presence of an almost constant number of reactions (501, on average) that are required to sustain life across all the time points examined. Interestingly, this set of reactions resembles, in percentage, the size of the minimal metabolic network predicted to be active in *E. coli* [37] (37% and 28% of the reactions embedded in the corresponding models).

To maintain such a constant trend, however, a deep reprogramming of the whole cellular metabolism appears to be necessary during the entire growth period, as up to more than 400 biochemical reaction, display at least one change in the predicted carried flux. According to our simulations, these changes do not only involve peripheral metabolic pathways (e.g. amino acid catabolism) but also a number of central pathways, e.g. TCA cycle, glycolysis and PP pathway. TCA cycle, for example, displays an increasing trend in the number of flux-carrying reactions parallel to the exhaustion (in the medium) of the amino acids whose degradation provide key TCA cycle intermediates. This is in line with the assumption that fluxes distribution within the cell are influenced by the entry-point of the a given C-source into the metabolic network [38]. Indeed, the C sources provided in our simulations have different distances from the *Ph*TAC125 central metabolism (e.g. TCA cycle) and thus are expected to cause a re-wiring of an important fraction of the network.

This, in turn, suggests the presence of an efficient metabolic reprogramming machinery (that includes the regulation of the expression of the corresponding genes) to continuously and promptly adapt to this nutritionally changing environment and/or to the exhaustion of the preferred carbon source(s). Modelling and dividing the growth curve in discrete time points has allowed us to infer common trends in the predicted flux patterns of the reactions of the model. We have shown that such coupled patterns likely correspond to reactions and enzymes that work in a concerted fashion and that, in some cases, represent functional partners and/or be encoded by co-regulated genes. The modelling framework we have set up here has allowed gaining insights on the (co-)regulation of *Ph*TAC125 metabolic genes and, as in the case of the Arg regulon, to expand the current knowledge on commonly regulated genes. Furthermore, we have assembled a dataset of putatively co-regulated genes that can be used for further manual curation and/or subject to experimental validation.

Despite FBA-based predictions led to the identification of biologically consistent trends, the nutritional-MOMA approach we have implemented here suggests that care needs to be taken when choosing the objective function for constraint-based metabolic modelling. Indeed, when we accounted for the possibility that cells could be in a near-optimal or sub-optimal state (i.e. minimizing the reprogramming required at each nutritional transition) the two modelling frameworks predicted quite a different topological and functional reprogramming of *Ph*TAC125 cells. As it is currently not possible to unambiguously discern among these two alternative solutions provided by FBA and nutritional-MOMA, further experimental evidence (e.g. time-resolved transcriptomics) of *Ph*TAC125 cells grown in complex medium will allow deriving a clearer picture of those pathways that are really active during the growth and, consequently, which of the two approaches outperforms the other.

## Additional files

Additional file 1: The metabolic reconstruction and the Matlab codes used in this work. (ZIP 3936 kb)
Additional file 2: Additional analyses on the *Ph*TAC125 metabolic reconstruction showing i) the effect of expressing fluxes as a fraction of the biomass production flux and ii) number of active reactions in each PhTAC125 metabolic pathway following the nutrients switching. (PDF 457 kb)
Additional file 3: The list of the reactions and genes embedded in all the clusters identified through flux correlation analysis. (ZIP 79 kb)
Additional file 4: The whole set of data related to the identified motifs and their annotations (before manual curation), for each probed database. (XLSX 21 kb)

## Abbreviations

FBA: Flux balance analysis
FVA: Flux variability analysis
MOMA: Minimization of metabolic adjustment
*Ph*TAC125: *Pseudoalteromonas haloplanktis* TAC125
TCA: Tricarboxylic acid
TF: Transcription factor
