# Supplementary material for: Modelling microbial metabolic rewiring during growth in a complex medium
Source: BMC Genomics. 2016 Nov 24;17:970. doi: 10.1186/s12864-016-3311-0 (PMC5121958; doi:10.1186/s12864-016-3311-0)
Supplement: Additional file 2: — Additional analyses on the PhTAC125 metabolic reconstruction showing i) the effect of expressing fluxes as a fraction of the biomass production flux and ii) number of active reactions in each PhTAC125 metabolic pathway following the nutrients switching. (PDF 457 kb) [file 12864_2016_3311_MOESM2_ESM.pdf]

Additional File S2

Modelling microbial metabolic rewiring during growth in a complex medium

Marco Fondi*§, Emanuele Bosi*, Luana Presta, Diletta Natoli, Renato Fani

Dep. of Biology, University of Florence, Via Madonna del Piano 6, I-50019 Sesto F.no, Italy,

** These authors contributed equally*

§ Corresponding author

Expressing fluxes as a fraction of the biomass production flux

To account for the influence of the growth rate over the computed fluxes distribution, we have expressed the predicted reaction fluxes across all the time points as a fraction of the predicted growth rate in the corresponding time point. More in detail, for each of the growth phases (*j*), all the fluxes have been expressed as:

$$f_{N,i} = \frac{f_i}{\mu_j * 10}$$

Where $f_{N,i}$ is the normalized flux of the i_{th} reaction, f_i is the originally predicted flux of the same reaction and μ_j is the predicted *Ph*TAC125 growth rate in in the j_{th} growth phase (with *j*=1:10).

Results obtained (Figure S1) revealed no major differences in the overall trends of the number of reactions carrying and changing flux in each phase and flux increase/decrease patterns compared to the original calculation of fluxes distribution (Figure 1 C-E in the main text).

In particular, as expected, the number of flux-carrying reactions remains the same as in the original simulation whereas slightly different values were obtained for i) reactions changing their flux across all the transitions and ii) the ratio between flux-increasing and flux-decreasing reactions. Nevertheless, in the first case, transitions T4, T5 and T8 were confirmed as the most demanding in terms of metabolic readjustments as in the original simulations (Figure 1D, main text). Similarly, albeit with an exaggerated oscillation among the 10 growth phases, the proportion between flux-increasing and decreasing reactions is maintained.

Overall, these data suggest that our results hold true regardless of the normalization procedure adopted to account for the different (decreasing) growth rates imposed to the model across the growth period.

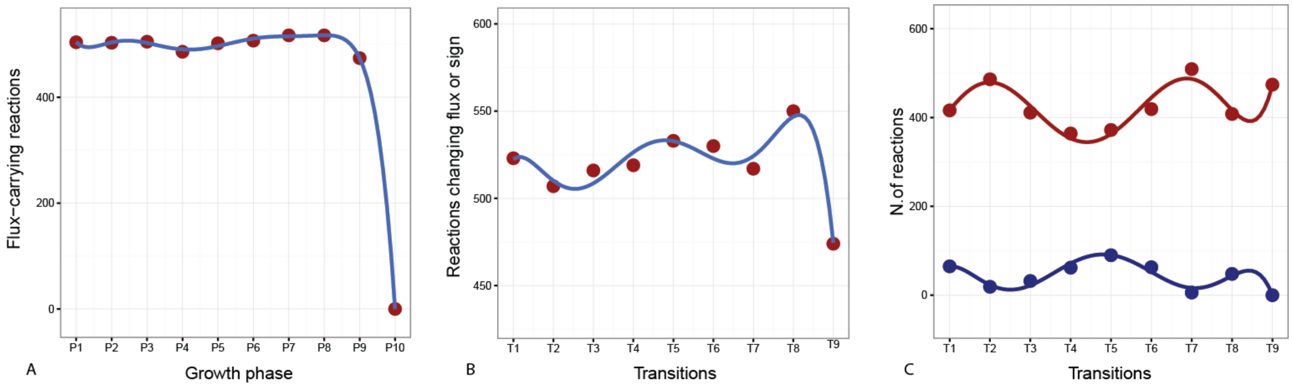

Figure S1: Summary of PhTAC125 genome-scale reprogramming following nutrients switching expressing fluxes as a fraction of the biomass production flux. a. Number of flux carrying reactions in each growth phase. **b.** Number of flux-changing reactions in each growth phase. The dashed line represents the average number of reactions carrying flux over all time points. **c.** Number of reactions whose flux is predicted to increase (blue line) and decrease (red line) following each shift in the nutrients provided.

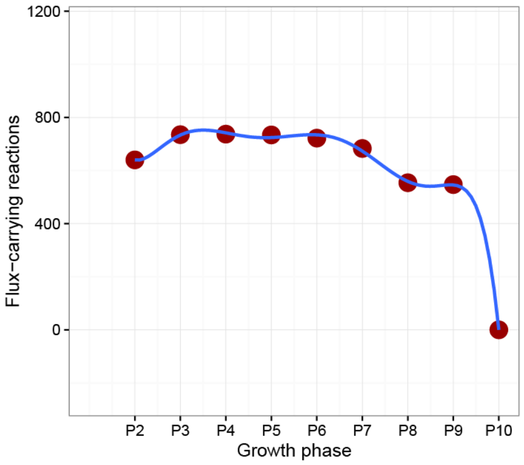

Figure S2: Number of flux carrying reactions in each growth phase performing a MOMA optimization on the *Ph*TAC125 model. Here, the number of predicted flux carrying reactions in each growth phase is shown when the model was optimized minimizing the number of metabolic adjustments required when switching among the different nutrient sets.

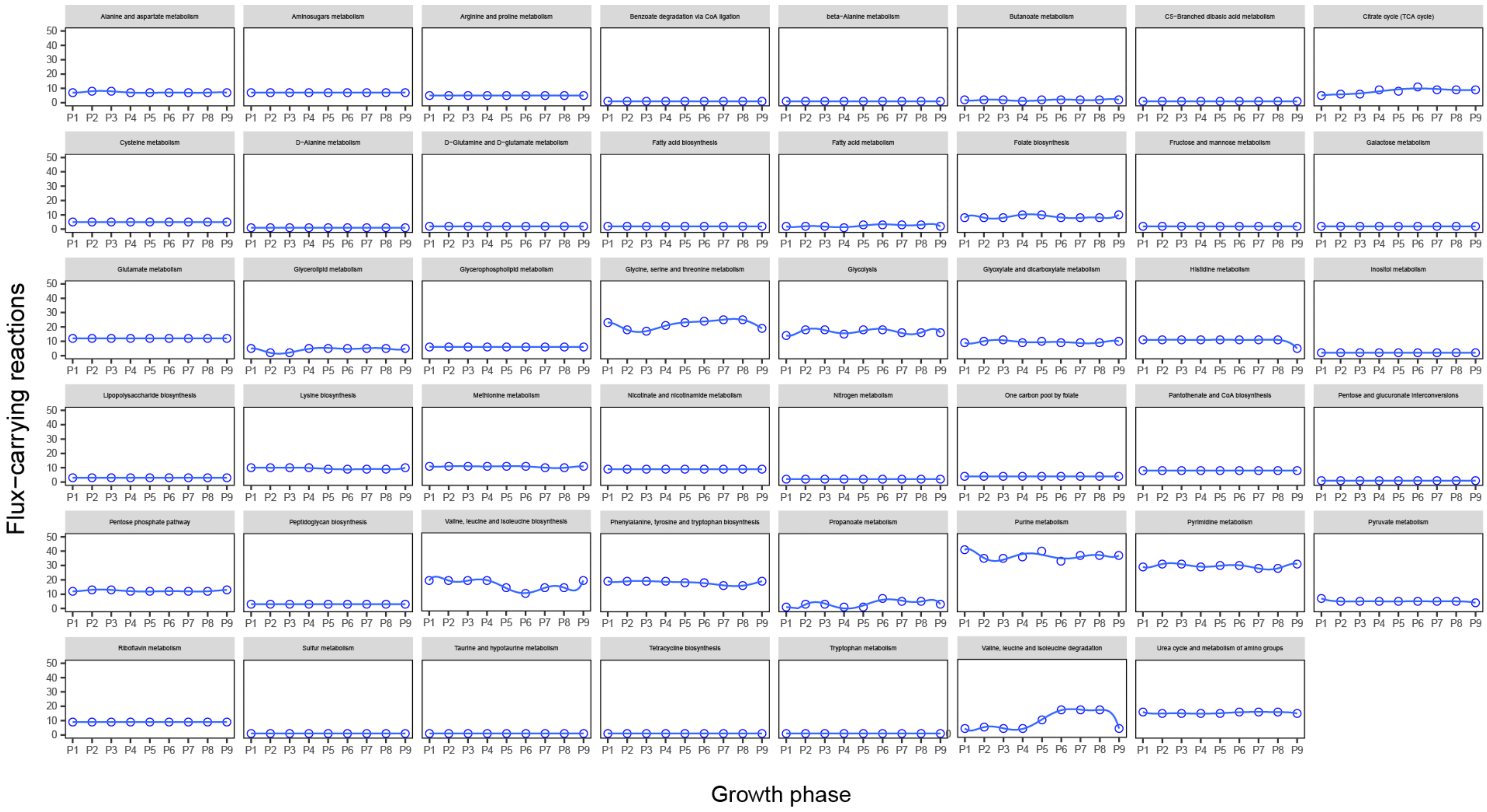

Figure S3: Number of active reactions in each PhTAC125 metabolic pathway following the nutrients switching
